# Intracardiac Thrombus in Coronavirus Disease-2019

**DOI:** 10.7759/cureus.22883

**Published:** 2022-03-06

**Authors:** Suganya Karikalan, Munish Sharma, Megha Chandna, Manju Sachdev, Ajay Gaalla, Farah Yasmin, Reena Shah, Iqbal Ratnani, Salim Surani

**Affiliations:** 1 Medicine, Karpaga Vinayaga Institute of Medical Sciences, Chennai, IND; 2 Internal Medicine, Baylor College of Medicine, Houston, USA; 3 Medicine, Texas A&M University, College Station, USA; 4 Pediatrics, Texas A&M Health Science Center, College Station, USA; 5 Medicine, DeTar Hospital, Victoria, USA; 6 Internal Medicine, Dow University of Health Sciences, Karachi, PAK; 7 Medicine, Aga Khan University, Nairobi, KEN; 8 Critical Care Medicine, DeBakey Heart & Vascular Center, Houston, USA; 9 Anesthesiology, Mayo Clinic, Rochester, USA; 10 Clinical Medicine, University of Houston, Houston, USA

**Keywords:** right ventricular thrombus, cardiomyopathy, covid-19 and cardiomyopathy, ventricular thrombus, corona virus disease, covid-19

## Abstract

Intracardiac thrombus is often seen as a complication of ischemic heart disease (IHD) and non-ischemia cardiomyopathies (NICM). The advancements in imaging modalities and therapeutic options have helped reduce the complications arising from ventricular thrombi, such as systemic embolization. Here we present two cases of intracardiac thrombus associated with coronavirus disease (COVID) 19, one with an apical thrombus in the left ventricle and the other with a thrombus in the right ventricle adjacent to chordae tendinae. The effects of covid-19 on the cardiovascular system are yet to be thoroughly evaluated. Venous and arterial thrombosis is commonly associated with COVID-19 but in situ detection of intracardiac thrombus has not been very frequently reported. Intracardiac thrombus and embolization pose a very high risk of complications in COVID-19.

The coronavirus pandemic caused by SARS-CoV-2 during 2019-2021 has caused several deaths and has resulted in many long-term consequences, many of which remain unclear. In-hospital complications from COVID-19 are better reported due to constant monitoring. The ongoing, late, and chronic complications arising from COVID-19 require more vigilant case-by-case screening and surveillance.

## Introduction

The incidence of left ventricular thrombus is 15% in patients with ST elevated myocardial infarction (STEMI) and 2-36% in patients with non-ischemic cardiomyopathies. The risk of systemic embolization increases mortality and morbidity regardless of the etiology of the thrombus [1]. The advent of thrombolytic agents has reduced the incidence of thrombus post-acute phase of myocardial infarction. The spectrum of cardiovascular diseases in the COVID-19 range from myocardial injury to various cardiomyopathies and thromboembolic events. The mechanisms resulting in these events include cytokine storm, virus-mediated damage to organs, prothrombotic states, hypoxia, and the long duration of illness and its complications. Though many complications of COVID-19 such as arterial and venous thromboembolism, cardiomyopathy are reported across multiple articles, only a few cases of intracardiac thrombus due to COVID-19 have been reported per se.

## Case presentation

Case 1

**Figure 1:**
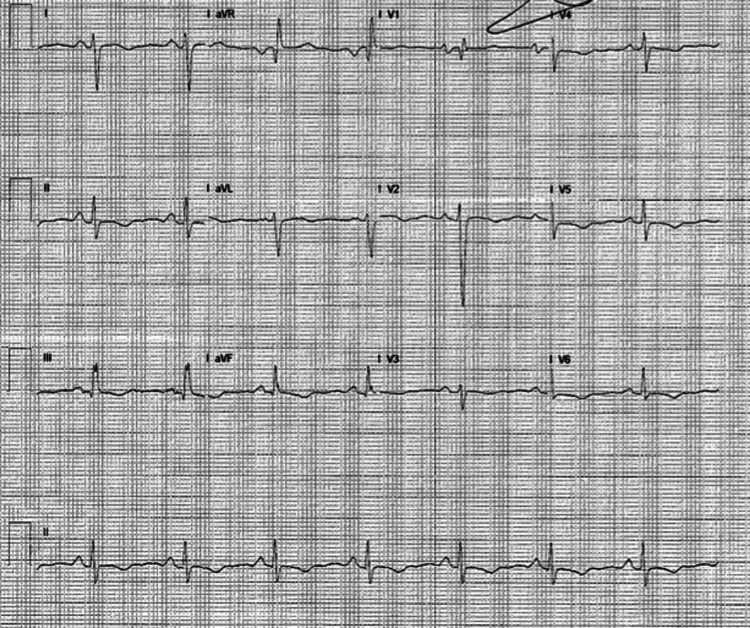
ECG showing normal sinus rhythm with right bundle branch block

A 43-year-old female patient with a history of hypertension and diabetes mellitus presented to the emergency department with complaints of dizziness, headache, increasing shortness of breath, dry cough, weight gain, and leg swelling. The patient had a history of COVID-19 six months prior to this presentation, which was diagnosed by the polymerase chain reaction (PCR). On examination, she was dyspneic, non-hypoxic, with a respiratory rate of 30/minute, blood pressure 176/110 mmHg, heart rate of 95 beats/minute. She had bilateral pitting pedal edema. Cardiac examination revealed S1 S2 with S3 heard on auscultation. Chest examination revealed crackles in the bilateral lung bases. She was started on supplemental oxygen and intravenous diuretics. Chest X-ray showed interstitial infiltrates. Electrocardiogram showed normal sinus rhythm with right bundle branch block (Figure 1).

**Figure 2:**
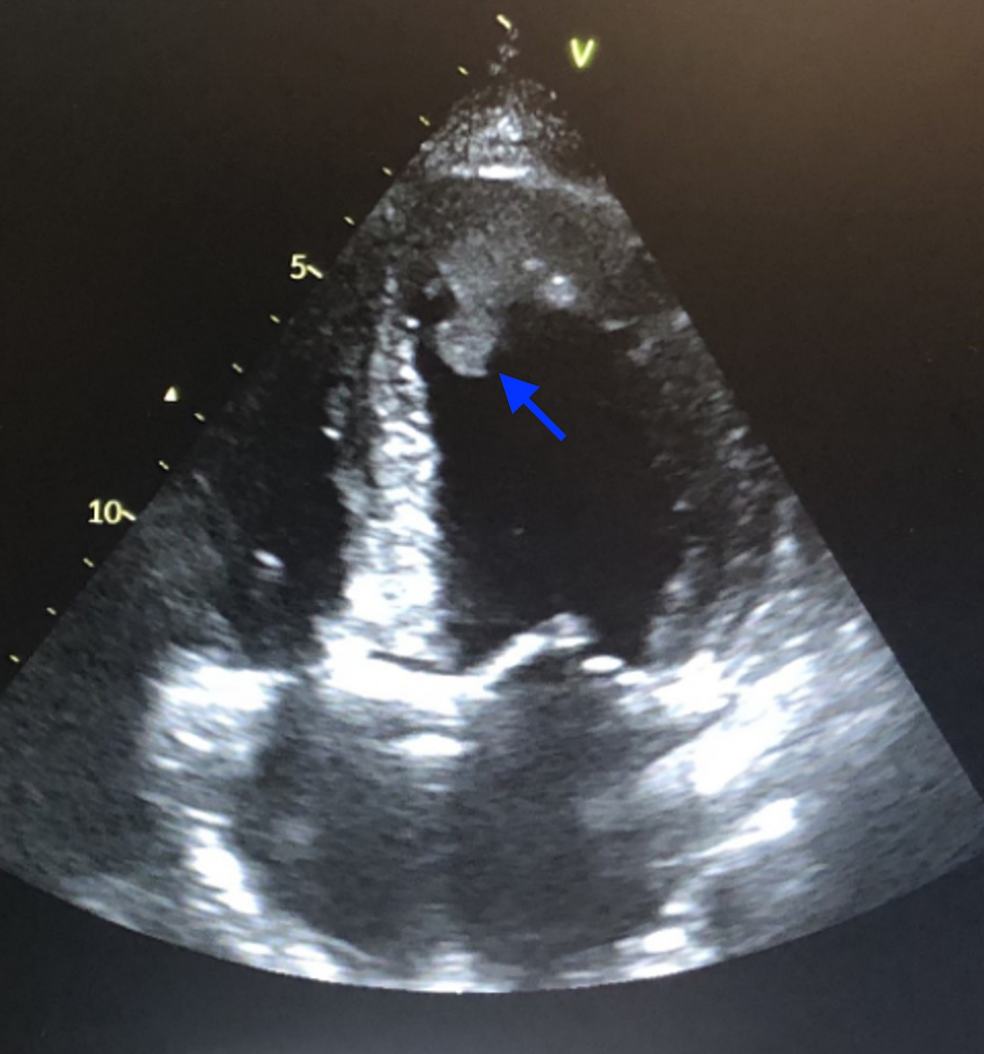
Echocardiogram showing globally enlarged ventricles with an apical thrombus in the left ventricle (blue arrowhead)

Echocardiography showed a globally reduced left ventricular ejection fraction of 25% and a left ventricular apical mural thrombus of 1.8cm (Figure 2). A computed tomography scan of the brain showed evidence of acute right temporal and occipital infarcts, left posterior parietal or watershed infarct in the left temporal insula, findings suggestive of embolic thromboembolism. The patient was diagnosed with non-ischemic cardiomyopathy secondary to COVID-19 with subsequent LV thrombus and emboli to the brain. The patient was treated with antiplatelets, heparin, and antihypertensive medicines. The patient improved symptomatically during the hospital course on the medical floor and was discharged home on antihypertensive medications, diuretics, beta-blockers, and direct oral anticoagulants (DOACs).

A repeat echocardiogram on outpatient follow-up after a month showed an improved LV ejection fraction of 45% and a 1.5cm mass at the LV apex. Her congestive heart failure continued to show improvement since her discharge. Hence, beta-blockers and diuretics were continued with oral anticoagulation for left ventricular thrombus.

Case 2

**Figure 3:**
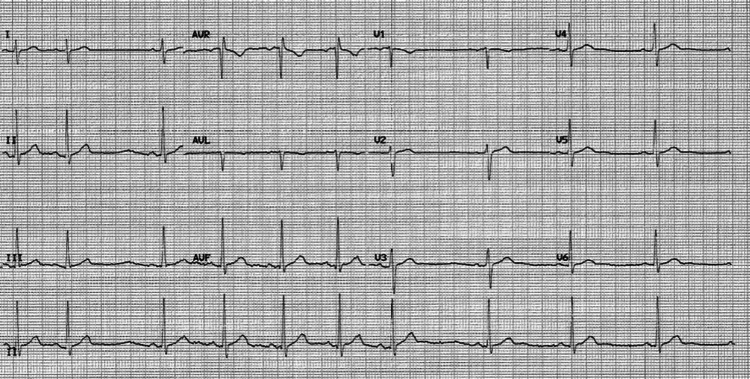
ECG showing normal sinus rhythm, no ST-T changes

A 33-year-old male presented with complaints of shortness of breath on exertion and palpitations. The patient had a history of COVID-19 pneumonia two months prior to this presentation via PCR testing. On examination, the patient was afebrile, heart rate of 84 beats/min, regular, and blood pressure of 140/90 mmHg. On auscultation, S1 and S2 were regular in rhythm, and no S3 was heard. Abdomen, respiratory and neurological examination was normal. EKG showed normal sinus rhythm (Figure 3).

**Figure 4:**
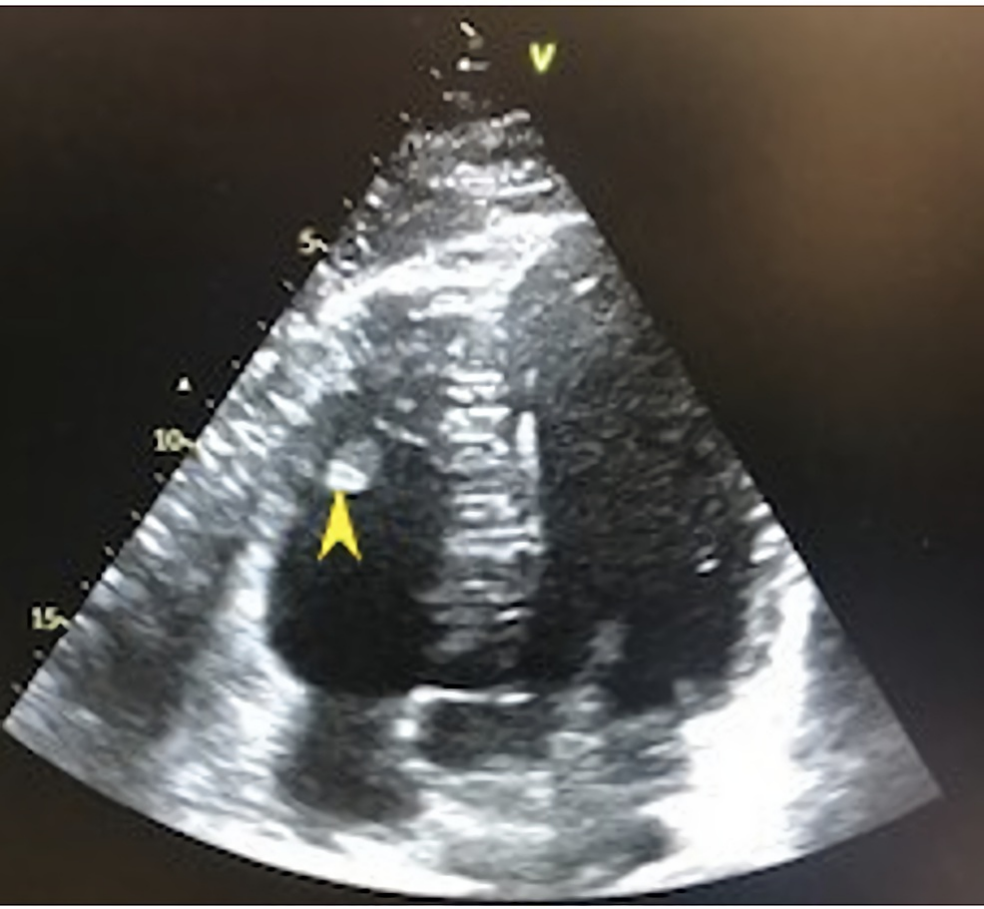
Echocardiogram showing clot in the right ventricle (yellow arrowhead)

An echocardiogram showed an ejection fraction of 60% with mitral and tricuspid regurgitation, enlarged right ventricle, and a 1.3-cm mobile mass attached to the tricuspid valve chordae tendinae (Figure 4).

**Figure 5:**
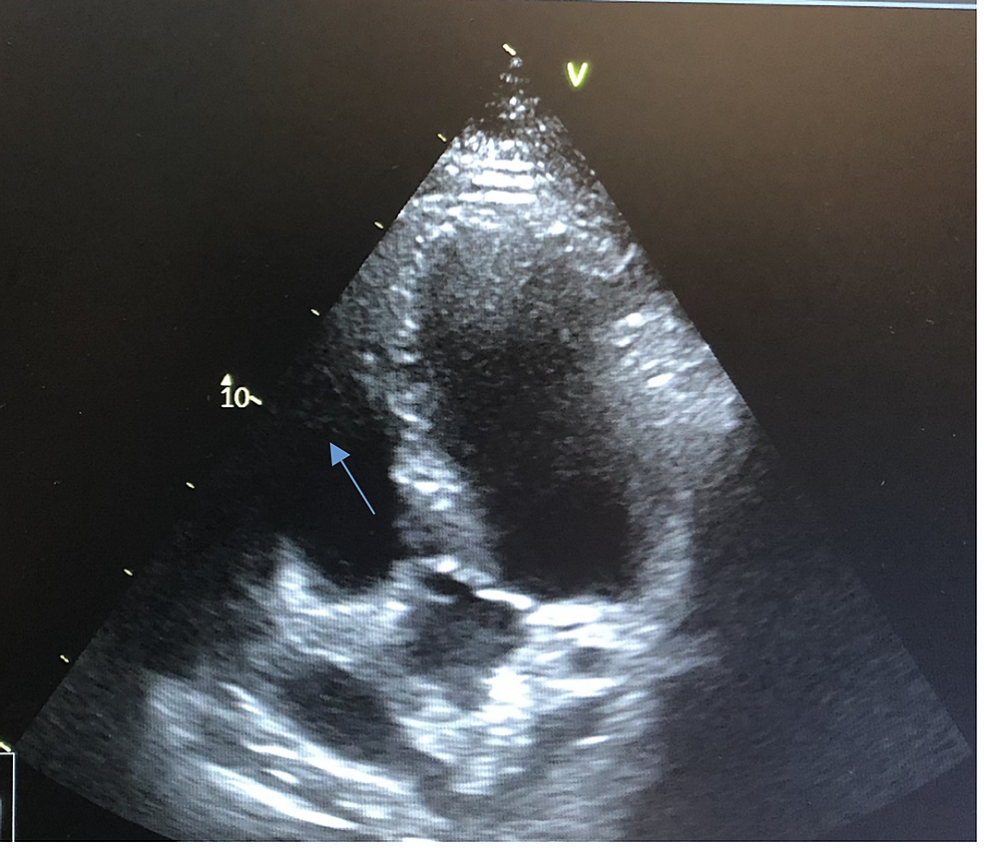
Echocardiogram showing no thrombus in the RV

The right atrial pressure was 5 mmHg, and the right ventricular systolic pressure was 35 mmHg. There was mild tricuspid regurgitation. The patient was started on diuretics with improvement in her dyspnea. He was also started on direct oral anticoagulants. The patient showed improvement in symptoms and was discharged home on oral apixaban on day 4 of hospitalization. The follow-up echocardiogram in three months showed resolution of the thrombus, right atrial pressure was 5 mmHg, and right ventricular pressure was 20 mmHg (Figure 5).

## Discussion

Left ventricular thrombus (LVT) without myocardial infarction

Intracardiac thrombus is commonly seen after acute MI or non-ischemic cardiomyopathy with severely reduced LV ejection fraction. With the advent of primary coronary intervention, the incidence has declined from 40% to 5-15% [1, 2]. The other causes of ventricular thrombus include myocarditis, cardiomyopathy, hypercoagulable states such as protein S and protein C deficiency, antiphospholipid syndrome, Behcet's disease, and blunt chest trauma. The incidence of left ventricular thrombus in patients with non-ischemic cardiomyopathy was found to be 2 to 36% [1]. Younger age, reduced ejection fraction, increased regional wall motion, lesser deceleration time, increased early diastolic filling velocity, and high left atrium area contributes to LVT in dilated cardiomyopathy. In stress-induced cardiomyopathy, the incidence of LVT was found to be 3-5.3%, the reason for the lower incidence being the transient nature of the disease. The apex is the most commonly involved region in the left ventricular thrombus in both MI and non-ischemic cardiomyopathy patients [3]. Biventricular systolic dysfunction in dilated cardiomyopathy results in biventricular stasis and can lead to thrombus formation in both the right and left ventricles, left being more common than right [3]. In non-ischemic cardiomyopathy patients with LV thrombus, the frequency of thrombus in other cardiac chambers is 40% higher than patients with Ischemic cardiomyopathy [4]. LV thrombus is associated with an increased risk of peripheral embolization. The trouble arises when the thrombus protrudes into the ventricular cavity or when the thrombus is highly mobile [5]. Such as the case in our patient with left ventricular thrombus, where it embolized to the brain, causing an embolic stroke. The mortality rate is higher in patients with peripheral embolization than patients with severe ventricular dysfunction without embolization [5]. In all patients with reduced ejection fraction, screening for LVT should be prioritized considering the embolic event and mortality risk.

Right ventricular thrombus is less frequent than LVT. The most common cause is deep vein thrombus. The right ventricular thrombus is often said to be ‘in transit since they originate from the deep venous system and travel from the periphery. Other causes include heart failure, intracardiac devices, and atrial fibrillation [6]. RV thrombus can be of three types: Type A - serpiginous highly mobile mass within right atrium or ventricle, Type B - non-mobile, mural, and Type C - similar to myxoma and highly portable. Type A is more common and is most often associated with acute pulmonary embolism [7]. Fortunately, our patient presented early and was diagnosed and treated, preventing the embolization of the right ventricular thrombus and causing pulmonary embolism.

Trans-thoracic echocardiography (TTE) is the recommended screening tool for thrombus detection. The TTE can be technically challenging due to patients’ body size, chest deformities, lung diseases, and small intercostal spaces. Though transoesophageal echocardiography (TEE) is considered superior to transthoracic echocardiography, its role in LVT is limited. The use of echocardiographic contrast agent (ECA) and harmonic imaging can improve the sensitivity and specificity of echocardiography. Cardiac magnetic resonance (CMR) imaging is superior to all other imaging modalities. It facilitates the detection of intraventricular structural abnormalities, scarred myocardium, etc.; yet, the higher cost and lesser availability pose a hurdle for CMR usage even in developed countries. Computerized tomography (CT) for LVT has the same accuracy as TTE, the only cons being increased radiation exposure and iodine contrast agent usage.

According to the guidelines, the treatment of choice for left ventricular thrombus is warfarin for a minimum of three to six months [8]. The use of warfarin requires monitoring of the international normalized ratio, food and drug restrictions due to interactions, and the use of bridging therapy to overcome the prothrombotic effect of vitamin K antagonist and the increased risk of bleeding [2]. The newer direct oral anticoagulants are being used across a wide scale due to the advantages such as less monitoring, fewer interactions, and the absence of a requirement of bridge therapy. The American Heart Association guidelines recommend the usage of low molecular weight heparin (LMWH), dabigatran, rivaroxaban, or apixaban for patients who are intolerant to warfarin therapy. Surgical thrombectomy is considered in hemodynamically unstable patients or patients undergoing open-heart procedures where thrombus poses a high risk for embolism. The mortality and morbidity associated with surgical thrombectomy outweigh the benefits of performing surgery for the sole purpose of ventricular thrombus [1].

The right ventricular thrombus is often associated with pulmonary embolism. It is known to originate from deep vein thrombus or in-situ due to cardiomyopathy or atrial fibrillation due to stagnation of blood [9]. There are no definitive guidelines for the management of right heart thrombus. Decisions are taken based on case-by-case scenarios and are often followed as per pulmonary embolism protocols. Initial management includes oxygen therapy, administration of right ventricular failure if present, followed by anticoagulation [10]. In hemodynamically stable patients, anticoagulation is initiated with unfractionated heparin, or low molecular weight heparin, followed later by oral anticoagulation. In case of hemodynamic severity or instability thrombolysis, surgical embolectomy is chosen on a case-by-case basis. In patients with recurrence and patients not suitable for medical and surgical treatment, the inferior vena cava (IVC) filter can be considered. As our patient was hemodynamically stable, he was started on LMWH and discharged with oral anticoagulants [10].

COVID-19 and hemostatic complications

The outbreak of COVID-19, caused by the SARS-CoV-2 virus, has affected more than 300 million people worldwide and caused approximately 5.5 million deaths. Despite being a primary lung disease, the cytokine storm causes an imbalance between T-cells and interleukin-6 (IL-6), IL-17, and other cytokines resulting in multiple organ damage [11]. The hemostatic complications such as ischemic stroke, deep vein thrombosis, acute pulmonary embolism seem to be higher in COVID-19 patients than non-COVID-19 patients [12]. Angiotensin-converting enzyme receptors are present in multiple organs. The SARS-CoV-2 virus binds to the ACE receptors, triggering endothelial dysfunction, which sets in recruitments of inflammatory mediators resulting in activation of the complement system. Prolonged bed rest due to hospital admission causes blood stasis. This results in thrombocytopenia, long prothrombin time (PT), international normalized ratio (INR), and thrombin time (TT). Another pathophysiology is neutrophils extracellular traps (NET) released by neutrophils [11]. COVID-19 can begin with cardiovascular diseases or can cause exacerbation of pre-existing cardiovascular illness. The post covid cardiovascular sequelae include presentations such as coronary syndrome, heart failure, cardiomyopathy, myocardial infarction, arrhythmias, and venous thromboembolism.

The patients presented above have no evidence of infarction. Myocardial injury due to SARS-CoV-2 infection could have resulted in cardiomyopathy, resulting in stagnation of blood, which might have resulted in ventricular thrombus formation. Younger age, non-ischemic cardiomyopathy supported using direct oral anticoagulants for their management. The patient’s follow-up with left ventricular thrombus post-initiation of DOAC showed echocardiographic evidence of reduction of thrombus size from 1.8 cm to 1.5 cm. Risk stratification for venous thromboembolism should be carried out in patients with COVID-19, similar to non-COVID-19 patients, considering the risks of acute illness and prolonged immobilization. Post-discharge prophylaxis with low molecular weight heparin or DOACs can be provided based on risk assessment at the time of discharge.

## Conclusions

COVID-19, despite being a primary respiratory disease, is known to cause new-onset cardiovascular illness and complicate existing cardiovascular conditions. The severity of acute illness, the requirement of prolonged hospitalization, the inflammatory nature of SARS-CoV-2 predisposes to thromboembolic complications, both during the illness and post-discharge. Intracardiac thrombosis in COVID-19 patients is not a frequently reported entity and can have significant associated mortality and morbidity. Thus, long-term follow-up of these patients and data sharing across multiple countries can aid in a proper understanding of the pathophysiology, management, and prevention of this condition.
